# Report of on Operation for Removing a Foreign Body from beneath the Heart

**Published:** 1857-12

**Authors:** 


*Report of an Operation for Removing a Foreign Body from beneath the Heart.* By E. S. Cooper, A. M., M. D., of San Francisco.

If we mistake not, this is the report of a case which went the rounds of the newspapers some weeks ago; it now appears in an authentic form before the profession. The patient had received a breach-pin in the chest, which accident resulted from the bursting of an old gun; he was brought to Dr. Cooper seventy-four days after the accident, in a condition of extreme prostration, discharging copiously from the wound in the thorax, and subject to fits of suffocation so severe as to endanger life. With the patient's full consent to the desperate alternative, and in the presence of a number of distinguished medical men, Dr. Cooper, after removing large portions of carious ribs, and searching for more than three-quarters of an hour, succeeding in removing the foreign body from beneath the heart, on the vertebral column just to the right of the descending aorta. From this operation the patient has now recovered.

This is certainly one of the most remarkable and daring exploits of modern surgery, and though in many, if not most similar cases, the patient would not be likely to recover, yet this shows that in a young man, under favorable circumstances, even such desperate efforts as this, may be crowned with success. This case will remain a lasting monument to the skill and daring of Dr. Cooper. F.

